# Current status of emergency department attending physician ultrasound credentialing and quality assurance in the United States

**DOI:** 10.1186/s13089-016-0042-z

**Published:** 2016-05-26

**Authors:** Devjani Das, Monica Kapoor, Cara Brown, Afoma Ndubuisi, Sanjey Gupta

**Affiliations:** Department of Emergency Medicine, Northwell Health-Staten Island University Hospital, 475 Seaview Ave, Staten Island, NY 10305 USA; Department of Emergency Medicine, Northwell Health-Franklin Hospital, 900 Franklin Ave, Valley Stream, NY 11580 USA

**Keywords:** Emergency ultrasonography, Education, Survey, Credentialing, Quality assurance

## Abstract

**Background:**

The use of emergency ultrasonography (EUS) has gained much popularity in the past few decades, and is now a mainstay of diagnostic decision-making. This expanded use is now highlighting the substantial issue of individual hospitals in credentialing its emergency medicine attending physicians in EUS in the United States. This issue is also of importance as more hospitals are now requesting reimbursements for emergency ultrasounds. The objective of this study is to gain an understanding of how many emergency departments are currently credentialing its attending staff in EUS, what the internal structure and staffing are of these emergency departments, and how they are currently performing quality assurance of the ultrasounds performed.

**Methods:**

This was a cross-sectional, web-based survey sent to 160 ACGME-accredited EM residency programs from July 2013 to November 2013. The survey consisted of 23 questions regarding: (1) number of emergency medicine attendings on staff, (2) presence of an EUS fellowship, (3) quality assurance (QA) process, and (4) current US credentialing process.

**Results:**

There was a 50 % response rate. Fifty percent of the total respondents (*n* = 40) had an EUS fellowship program. Of the sites with an EUS fellowship, 36 had EUS fellowship-trained attendings. Of the sites without an EUS fellowship, 19 had EUS fellowship-trained faculty, *p* ≤ 0.0001. Sites with an EUS fellowship had a greater percentage of staff credentialed to perform EUS as compared to sites with no EUS fellowship, *p* = 0.0161. All sites with an EUS fellowship had EUS-credentialed attendings. In sites with an EUS fellowship, 35 conducted a formal QA of ED performed EUS scans versus 22 at sites without an EUS fellowship, *p* = 0.003.

**Conclusions:**

The survey results support hiring emergency attendings that have completed postgraduate training in emergency ultrasonography to aid in credentialing staff. This also seems to be helpful in completing a timelier QA of all ED ultrasounds.

## Background

The use of emergency ultrasonography (EUS) has gained much popularity, since it was first introduced in the 1980s and is now a mainstay of diagnostic decision-making in the emergency department [1]. In 2011, the Accreditation Council for General Medical Education (ACGME) Residency Review Committee (RRC) adopted an outcomes-based approach to residency accreditation in the United States. As part of this endeavor, EUS was deemed as one of the essential components in Patient Care, which is one of six core competencies for emergency medicine residency training. Since adoption of this mandate, every emergency medicine (EM) residency program must teach and assess ultrasound competency as part of its education [2]. As a result of this directive, improvements in US technology and research into new potential applications for US, the use of EUS has increased in the emergency department (ED). This expanded use is now highlighting the substantial issue of individual hospitals in credentialing its emergency medicine attending physicians in EUS in the United States. This issue is also of importance as an increasing number of hospitals is now requesting reimbursement for emergency ultrasounds.

Numerous organizations within EM have proposed guidelines on how to train physicians in EUS applications. Foremost among these are the 2008 American College of Emergency Physicians (ACEP) guidelines that recommend 150 ultrasound examinations in six specific applications [3]. However, several studies have shown that though there is an increase in EP training in EUS, there remains a wide variation in the type and extent of teaching in individual academic training programs [4, 5]. This variability in teaching and education presents a challenging situation for EDs to credential attending physicians in the use of EUS.

An extensive review of the current literature has shown that no standardized method of ED attending credentialing in EUS exists in the United States. Furthermore, credentialing is primarily an individual hospital function. This lack of a universal credentialing process in EUS may lead to a discrepancy in the delineation of privileges for a physician from hospital to hospital, i.e., a physician credentialed in performing ultrasound in one hospital may not be credentialed to do so in another hospital [6]. The objective of this study is to gain an understanding of how many emergency departments are currently credentialing its attending staff in EUS, what the internal structure and staffing are of these emergency departments, and how they are currently performing quality assurance of the ultrasounds performed.

## Methods

### Study design

**Fig. 1:**
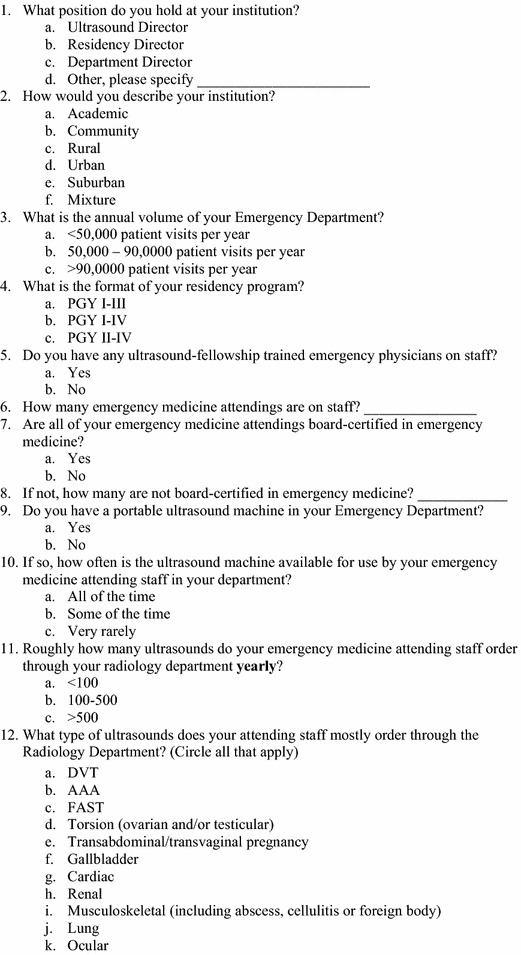
Survey questionnaire

**Fig. 2:**
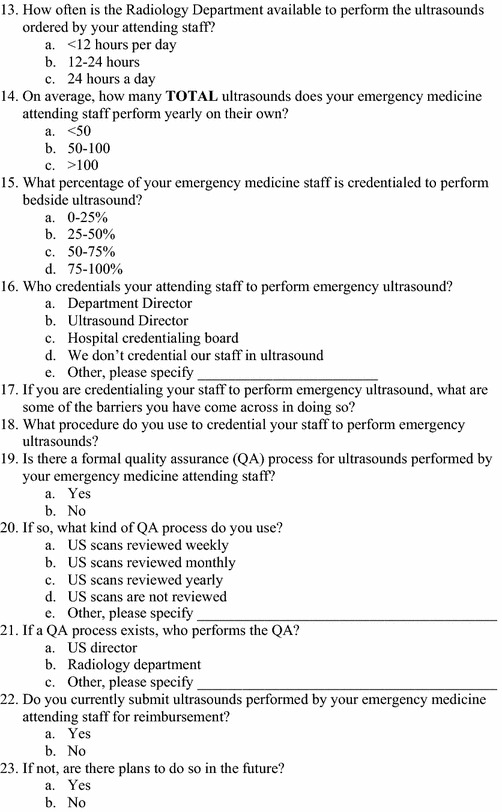
Survey questionnaire

This was a cross-sectional, web-based survey that was sent to 160 ACGME-accredited EM residency programs from July 2013 to November 2013. This was the total number of ACGME-accredited EM residency programs in existence during this time frame. A cover letter explaining the research study was initially sent to all EM residency program directors who were identified through the SAEM Residency Directory website. If deemed appropriate, the residency directors were instructed to share the survey with the US credentialing authority in their emergency department to acquire more accurate answers. A reminder email was sent 1 month after the initial survey request and again 2 months later to hospitals that had not completed the survey. The survey consisted of 23 questions created by the study authors regarding: (1) number of emergency attendings on staff, (2) presence or absence of an EUS fellowship, (3) quality assurance (QA) process, and (4) current EUS credentialing process (Figs. 1, 2). The survey required about 5–10 min to complete and included multiple choice and free text answers.

The survey was initially tested on a focus group consisting of three EUS program directors at various academic institutions. Questions were adjusted as suggested by the focus group for wording clarification purposes. Each academic institution that was sent a survey was provided with a unique numerical identifier after responses were obtained. All answers to the survey were de-identified and confidential prior to statistical analysis. This survey study was approved by the Institutional Review Board and was granted a waiver of signed consent at the authors’ home institution.

### Data analysis

The survey was distributed and results directly extracted from the online survey tool Surveymonkey©. Categorical data were analyzed utilizing descriptive statistics. The differences between groups were analyzed with the Chi-square analysis. Multivariate analysis was performed with a logistical regression model. Ordinal data were analyzed with a Mann–Whitney test and Spearman correlation.

## Results

There was a 50 % response rate to the survey study for a total of 80 respondents out of 160 programs (Table 1). All responses were from academic institutions in either a community (*n* = 22) or academic setting (*n* = 58). Of all respondents, 68.75 % (*n* = 55) had US-trained faculty on site. Fifty percent of total respondents (*n* = 40) had an EUS fellowship program. Of the sites with an EUS fellowship, 36 had EUS fellowship-trained attendings. Of the sites without an EUS fellowship, 19 had EUS fellowship-trained faculty, *p* ≤ 0.0001. Sites with an EUS fellowship had a greater percentage of staff credentialed to perform EUS as compared to sites with no EUS fellowship, *p* = 0.0161.

**Table 1:** Demographics of study population

Respondents	N (percentage)
Respondent demographics	
Number of respondents	80
Position in the institution	
Ultrasound director	23 (28.75)
Residency director	42 (52.5)
Department director	1 (1.25)
Other faculty	16 (20)
Institution description	
Academic	58 (72.5)
Community	22 (27.5)
Annual departmental volume (k visits)	
<50	6 (7.5)
50–90	35 (43.75)
>90	40 (50)
Residency program format	
PGY 1–3	53 (66.25)
PGY 1–4	26 (32.5)
EM/IM or EM/FM	1 (1.25)
Ultrasound demographics	
Does the site have portable ultrasound in the ED?	
Yes	80 (100)
No	0 (0)
Does the site have an ultrasound fellowship?	
Yes	40 (50)
No	40 (50)
Does the site have ultrasound trained faculty?	
Yes	55 (68.75)
No	25 (31.25)

### Ultrasound credentialing

Ultrasound credentialing was done by a variety of methods at different sites. All sites with an EUS fellowship had EUS-credentialed attendings. The designated EUS director usually performed the process of credentialing at these sites. At sites without an EUS fellowship, 7 had no credentialing for emergency attendings to perform EUS in the emergency department, *p* = 0.025 (Table 2).

**Table 2:** Credentialing and quality assurance responses

Respondents	N (percentage)
Percentage of ED attendings credentialed for EUS	
0–25 %	15 (18.75)
25–50 %	22 (27.5)
50–75 %	12 (15)
75–100 %	31 (38.75)
Who credentials the staff to perform EUS?	
Department director	16 (20)
Ultrasound director	50 (62.5)
Hospital credentialing board	32 (40)
Do not credential	7 (8.75)
Other	1 (1.25)
Is there a formal QA process for EP performed US?	
Yes	57 (71.25)
No	23 (28.75)
If a QA process exists, who performs the QA?	
Ultrasound director	56 (98.25)
Radiology department	1 (1.75)
N/A	23

## Quality assurance

In sites with an EUS fellowship, 35 conducted a formal QA of ED performed EUS scans versus 22 at sites without an EUS fellowship, *p* = 0.003. At hospital sites with an EUS fellowship that conducted formal QA, 100 % (35) had the QA process performed by the EUS director. In the non-EUS fellowship sites, 18 had the QA performed by the EUS director, *p* = 0.019. At non-EUS fellowship sites without an EUS director who are conducting QA, 4 utilized the following for QA: (1) assigned faculty member, (2) program director/director of pediatric ED, (3) hospital EUS credentialing committee, or (4) radiology department. At hospitals with an EUS fellowship that conducted a formal QA, 29 conducted QA weekly or less, 5 conducted QA bi-weekly-to-monthly, and 1 conducted QA in another format. At non-EUS fellowship sites, 5 conducted QA weekly or less, 13 conducted QA bi-weekly-to-monthly, 4 performed QA in another format (including rolling, yearly, or quarterly), *p* < 0.001. (Table 2).

## Discussion

The response rate to this survey study was on par with other studies conducted on similar topics. What differentiates this data set from other studies was that 50 % of total respondents were affiliated with sites supporting EUS fellowship programs, while 50 % were not. This allowed for a greater understanding of the difference in the credentialing and QA process at hospital sites with and without an EUS fellowship.

The survey results support that there is a great deal of variation in the number of EUS-credentialed staff and method for credentialing emergency attendings in EUS. This variability seemed to depend largely on the presence or absence of an EUS fellowship program at the institution. Having an EUS fellowship and a dedicated EUS director seems to be associated with wider credentialing of attending staff. This is likely due to having a greater number of faculty members to provide the additional training required to credential staff. However, this does not necessarily apply to institutions that have EUS fellowship-trained faculty, but no EUS fellowship on site. In addition, institutions without an EUS fellowship and without an EUS director are largely not credentialing their faculty to perform ultrasounds.

Similarly, there is variability in the QA process at hospital sites with and without an EUS fellowship program. Based on the results of the survey, hospital sites with an EUS fellowship are more likely to perform a formal QA process. Overwhelmingly, the majority of the hospitals manage their QA process within the ED. However, at hospital sites without an EUS fellowship program, there was greater variability in the method by which the QA process is currently being performed. Of hospital sites with a formal QA process, ultrasounds are likely to be reviewed on a weekly basis. However, if no EUS fellowship program is present, there is a greater likelihood that the QA process is not being conducted on a weekly basis. However, studies have shown that a weekly QA process is the optimal method to provide feedback, performance improvement, and assurance that ultrasounds are being performed and interpreted correctly [7].

In contrast to other medical specialties that utilize ultrasound, the examinations performed by emergency physicians are highly focused, limited, and goal-directed [8]. As Jang, et al. put forth in his recent article regarding competency-based mandate in EUS, to be considered competent in EUS, physicians must have (1) an understanding of the disease process and indications for the diagnostic modality, (2) technical skills to acquire appropriate and interpretable images, (3) the ability to reliably interpret sonograms, and (4) management skills to apply the findings in light of their patient’s clinical presentation [8]. The American Board of Emergency Medicine (ABEM) has been seeking to gain approval amongst its members to offer physician certification in clinical ultrasonography through the American Board of Medical Specialties (ABMS). ABEM envisions that once this is approved, physicians who do not achieve certification in clinical ultrasonography might eventually have a more difficult time practicing or billing for ultrasonography in some institutions. However, the process by which this may occur will require rigorous review through ABMS and may not occur for several years. As this move by ABEM highlights, it is becoming increasingly apparent that EUS is a vital skill in the ED management of patients. Currently, there exist evidence-based recommendations and expert consensus recommendations for specific applications for EUS, including for point-of-care lung and cardiac ultrasound [9, 10]. There have also been well-researched and comprehensive proposed guidelines/curriculum for teaching EUS and providing a foundational knowledge of point-of-care ultrasonography. However, these guidelines have not been prospectively validated [11]. Therefore, training and assessment of ultrasound education among different residency programs vary greatly [12]. What this study poses is the understanding that having an EUS fellowship, or at least staff that have completed an EUS fellowship, is associated with increased training of attendings in emergency ultrasonography.

Another key factor that was highlighted in this study was the variability in which hospitals are currently performing their QA process. As hospitals are moving forward with more widespread use of EUS, many hospitals are already, or are soon planning, to request reimbursement for ultrasounds performed in the ED. Each US that is performed, used to make clinical decisions and are subsequently submitted for reimbursement require appropriate documentation regarding the indication for the examinations, written interpretation of the examinations, description of the organs or structures studied, who performed the examinations, and documentation of the scope of the study [13]. For patient safety purposes, it is, therefore, imperative that all of these studies be reviewed for QA on a timely basis. Having an EUS fellowship is likely conducive to having additional staff to review all of these ultrasounds and to ensure that the QA process is more streamlined.

### Limitations

There are inherent limitations to any study based on survey results. This survey was subject to selection bias in that hospital sites that regularly utilize EUS are more likely to respond to the questionnaire. However, our response rate allowed for a unique data subset to analyze given that exactly 50 % had an EUS fellowship program versus 50 % that did not.

This study was also limited because hospital sites were chosen exclusively for having an ACGME-accredited EM residency program. This was done so that a greater understanding of the credentialing and QA process could be made at sites that are likely to be invested in having a robust EUS education program. However, results may be quite different if applied to hospital sites without an EM residency program and may not be generalizable to non-academic institutions.

## Conclusions

The spectrum of growth of EUS in daily practice within EDs nationwide is evident over the past several decades. This was exemplified by the RRC, which has made US education one subcomponent of its six emergency medicine core competencies. It is clear, therefore, that all hospitals, especially those with an ACGME-accredited residency program, will need to ensure that its staff is well versed in their US knowledge and technical skills. Credentialing their emergency attendings in EUS is a hospital-specific issue, but is a complex one when considering the extensive requirements that all physicians must meet to be considered competent in emergency ultrasonography. Our survey results seem to support hiring staff that has completed extra training in emergency ultrasonography to aid in credentialing other staff in EUS. This also seems to be helpful in completing a more timely QA process of all ultrasounds performed in the ED. In the future, we hope to look into what methods hospitals are currently using to credential their staff in EUS and which of these methods appear to be most effective.

## Abbreviations

EUS: emergency ultrasonography
ACGME: Accreditation Council for General Medical Education
RRC: Residency Review Committee
EM: emergency medicine
ED: emergency department
ACEP: American College of Emergency Physicians
